# Dual role of TRBP in HIV replication and RNA interference: viral diversion of a cellular pathway or evasion from antiviral immunity?

**DOI:** 10.1186/1742-4690-2-65

**Published:** 2005-10-27

**Authors:** Anne Gatignol, Sébastien Lainé, Guerline Clerzius

**Affiliations:** 1 Virus-Cell Interactions Laboratory, Lady Davis Institute for Medical Research, and Department of Medicine and Microbiology & Immunology, McGill University, Montréal, Québec, Canada

## Abstract

Increasing evidence indicates that RNA interference (RNAi) may be used to provide antiviral immunity in mammalian cells. Human micro (mi)RNAs can inhibit the replication of a primate virus, whereas a virally-encoded miRNA from HIV inhibits its own replication. Indirect proof comes from RNAi suppressors encoded by mammalian viruses. Influenza NS1 and Vaccinia E3L proteins can inhibit RNAi in plants, insects and worms. HIV-1 Tat protein and Adenovirus VA RNAs act as RNAi suppressors in mammalian cells. Surprisingly, many RNAi suppressors are also inhibitors of the interferon (IFN)-induced protein kinase R (PKR) but the potential overlap between the RNAi and the IFN pathways remains to be determined. The link between RNAi as an immune response and the IFN pathway may be formed by a cellular protein, TRBP, which has a dual role in HIV replication and RNAi. TRBP has been isolated as an HIV-1 TAR RNA binding protein that increases HIV expression and replication by inhibiting PKR and by increasing translation of structured RNAs. A recent report published in the Journal of Virology shows that the poor replication of HIV in astrocytes is mainly due to a heightened PKR response that can be overcome by supplying TRBP exogenously. In two recent papers published in Nature and EMBO Reports, TRBP is now shown to interact with Dicer and to be required for RNAi mediated by small interfering (si) and micro (mi)RNAs. The apparent discrepancy between TRBP requirement in RNAi and in HIV replication opens the hypotheses that RNAi may be beneficial for HIV-1 replication or that HIV-1 may evade the RNAi restriction by diverting TRBP from Dicer and use it for its own benefit.

## 

RNA interference (RNAi) is a natural antiviral mechanism in plant and insect cells. It can also be induced by mammalian and insect viruses in *Caenorhabditis elegans*, although there is no worm-specific virus isolated so far. An increasing number of observations indicate that RNAi may also be used by mammalian cells to counteract virus infection as a natural innate immunity [1-6]. A large number of mammalian viruses have been downregulated *in vitro *and *in vivo *by RNAi using exogenous small interfering (si)-, short hairpin (sh)- or micro (mi)- RNAs, showing that mammalian cells have the potential to mediate RNAi and to inhibit viruses by this mechanism [7,8]. In addition to cytokine production and the interferon (IFN) response, higher eukaryotes may have developed the RNAi mechanism as an additional innate immune response to pathogen infection. Alternatively, cells may have adapted this ancient mechanism required for developmental regulation as a response to prevent invasion by exogenous nucleic acids.

Several pieces of evidence support the role of RNAi as an antiviral immune response in mammalian cells [5]. Viral miRNAs isolated from cells infected by Epstein-Barr virus (EBV) and HIV-1 constitute the first evidence of a role of the RNAi mechanism during viral infection [9,10]. Retroviruses provide another example showing that a cellular miRNA restricts the replication of the primate foamy virus PFV-1 in human cells [11]. Other indirect support for this hypothesis is the presence of virus-encoded RNAi suppressors. Influenza virus NS1 and vaccinia E3L proteins, two inhibitors of the IFN-induced protein kinase R (PKR), inhibit RNAi pathways in plants and in *Drosophila *cells [12]. HIV-1 Tat protein acts as an RNAi suppressor in the pathway mediated by shRNAs but not siRNAs, suggesting a specificity of action [10]. Adenovirus VA RNAI and VA RNAII are cleaved by Dicer and act as RNAi suppressors [13]. Both Tat protein and VA RNAs inhibit Dicer activity. A striking feature of RNAi suppressors characterized thus far from mammalian viruses is that most are also inhibitors of PKR, either by direct binding, by RNA sequestration or by substrate competition [14]. However, this feature is not shared by plant and insect silencing suppressors. This characteristic suggests a link or an overlap between the mechanism of RNAi and the PKR pathway in mammalian cells. One common feature is that both mechanisms are triggered by dsRNA, but three recent papers published in Nature, EMBO Reports and the Journal of Virology establish another link through a double-stranded (ds) cellular RNA binding protein, TRBP. TRBP binds Dicer and is part of the RNA-induced silencing complex (RISC), but it is also a strong inhibitor of PKR responsible for enhancement of HIV-1 replication [15-17].

TRBP was isolated as an HIV-1 trans-activation response (TAR) RNA binding protein that enhances virus expression [18,19]. It belongs to the family of dsRNA binding proteins with two dsRBDs and a KR-helix motif within dsRBD2 that mediates RNA binding. A third C-terminal basic domain does not mediate RNA binding [20,21]. TRBP is a strong PKR inhibitor by direct binding through its dsRBDs and by dsRNA sequestration [22-24]. TRBP also has a direct activity on translation independent of PKR but dependent on a structured RNA [25]. All assays done thus far with HIV show that the protein contributes positively to the enhancement of HIV-1 expression and replication (Fig. 1). A recent paper in the Journal of Virology further demonstrates this ability. In Ong *et al*., [17] published in the October 15 issue (chosen as a spotlight by the editors), the authors demonstrate that HIV-1 replicates poorly in astrocytes because of a heightened PKR response, that mediates poor translation of the viral structural proteins. They demonstrate that HIV replication can be rescued by expressing low amounts of the PKR inhibitor TRBP. In this context, TRBP prevents PKR activation, restores the production of viral proteins and consequently HIV replication. The profound impact of TRBP transfection in these cells comes from its low endogenous expression due to a weak activity of TRBP promoter [26]. The low permissivity to HIV replication in astrocytes can therefore be ascribed in large part to low TRBP expression. This recent paper provides an additional mechanistic explanation for the low HIV replication in astrocytic cells and demonstrates the key role of TRBP in virus translation by counteracting the antiviral immunity mediated by PKR.

**Figure 1:**
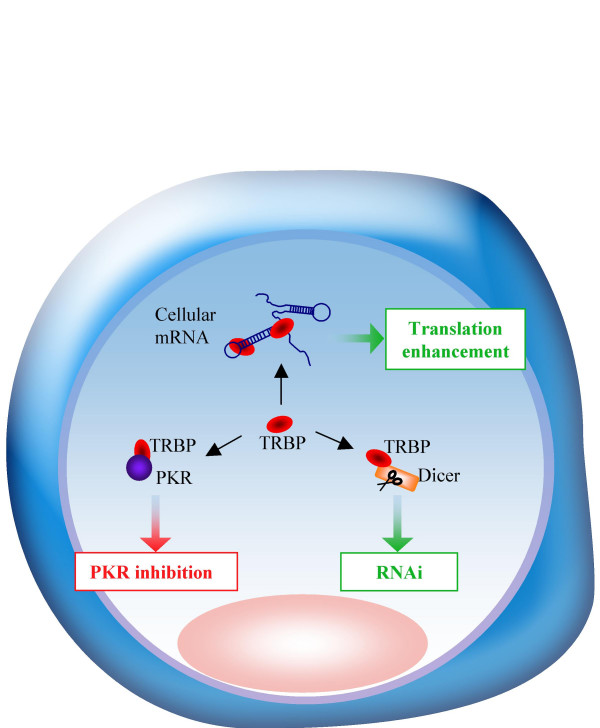
**TRBP acts in the cell by at least three different mechanisms: i) **it enhances translation by binding to dsRNAs; **ii) **it binds to PKR and inhibits its function;**iii) **it participates to the RNAi pathway by interacting with Dicer.

At the same time two papers published recently in Nature and EMBO Reports show that TRBP binds Dicer, that it is part of RISC, and that it is required for RNAi in human cells [15,16]. In both papers, the authors isolated ribonucleoprotein complexes containing Dicer, analyzed them by gel electrophoresis and mass spectrometry. Argonaute2 (Ago2) and TRBP were among the proteins found reproducibly in the complex. The interaction between TRBP and Dicer was confirmed by immunoprecipitation and *in vitro *interaction. Haase *et al*., show that the interaction is independent from RNA and that the complex cofractionates with the miRNA miR-17. By using a two-hybrid assay, they map the interaction to the C-terminal domain in TRBP, which is devoid of RNA binding activity, providing further evidence of a direct interaction between the two proteins. Chendrimada *et al*., show that TRBP forms ribonucleoprotein complexes composed of [siRNA-TRBP-Dicer-Ago2], indicating that TRBP appears as the bridge between dsRNA and Dicer for Ago2 recruitment. Using siRNAs against either TRBP or Dicer and subsequent immunoprecipitation of Ago2, the authors show a decrease in both TRBP and Dicer concluding to a loss of stability of the reciprocal partner. However, this decrease rather indicates that Ago2 requires both proteins to effectively bind the complex. Using the same siRNAs, they further show a decrease in mature miRNA production and a loss of siRNA function either after TRBP or after Dicer depletion. They conclude that TRBP recruits Ago2 to RISC and that it couples the initiation and the execution steps of RNAi. Haase *et al*., show that *in vitro *processing, but not *in vivo *steady-state levels of miRNAs, is decreased by TRBP depletion. SiRNAs against TRBP did not cause destabilization of Dicer, but decreased the efficiency of RNAi mediated either by miR17 or by an anti-lamin siRNA, indicating that TRBP is involved in both processes. The conclusion of both papers is that TRBP is a partner of Dicer that is required for siRNA as well as miRNA function in human cells.

Considering that TRBP is required both for RNAi and for an efficient HIV replication, it is difficult to understand how RNAi could function as a cellular antiviral mechanism against HIV. In this regard, two possibilities arise: 1) Instead of mediating antiviral immunity, RNAi could be beneficial for the virus and 2) HIV may divert TRBP and use it for its own benefit to avoid RNAi cleavage.

1) Could HIV replication benefit from the RNAi pathway? Because RNAi is a mechanism that cleaves RNAs homologous to defined siRNAs, it should participate to the elimination of unwanted exogenous RNA to protect the cell. However, numerous examples show that viruses also co-opt cellular pathways and use them for their own replication [14]. Therefore, we cannot exclude that the RNAi pathway can support HIV replication and possibly other viruses. Indeed, one recent study shows that a liver-specific miRNA, miR-122, facilitates hepatitis C virus (HCV) replication, although in this case, the virus and the cell may have co-evolved with miR-122 [27]. TRBP and Dicer may be recruited to the TAR RNA, to the Rev response element (RRE) RNA, to the virus-encoded (v)siRNA, or to other dsRNA parts of the entering virus to form ribonucleoprotein complexes. These complex formations would induce cleavage of dsRNA that would be beneficial for the virus. An argument against this hypothesis is the activity of vsiRNA, which is able to cleave the HIV envelope mRNA and inhibit virus replication when Tat is mutated [10]. In favor of this hypothesis is the positive activity of TRBP on HIV expression and replication, the ability of TRBP to bind TAR and RRE RNA, and the presence of short transcripts corresponding to the size of TAR RNA during viral infection. Although these transcripts likely stem from an ineffective transcription [28], it cannot be excluded that some are in fact generated by Dicer cleavage after TRBP binding to the 5' or the 3' TAR structure of the incoming virus. A large amount of TAR RNA in the cell inhibits PKR [29], and this may also be the case for other HIV small dsRNAs. This RNA-mediated inhibition would relieve the IFN innate immunity and favor virus replication. Alternatively, TRBP in RISC could favor the cleavage of cellular miRNAs that would favor HIV replication and the virus would have evolved in cells producing these RNAs. More studies on the relationship between the presence of short TAR RNAs and vsiRNAs, cellular miRNAs, the RNAi function and PKR activity during the viral replication cycle will be needed to evaluate this hypothesis (Fig. 2A).

**Figure 2:**
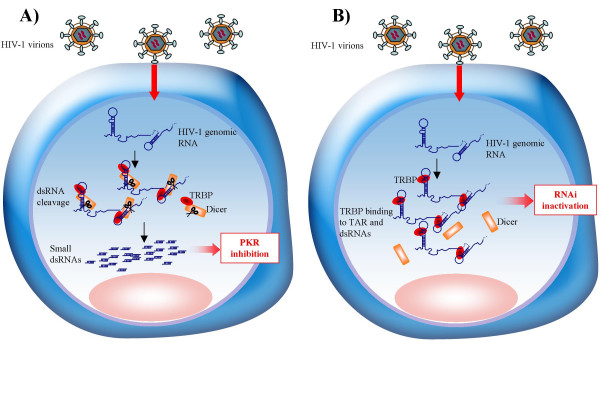
**Model for the role of TRBP during the early steps of viral infection. A) HIV co-opts the RNAi pathway for its own benefit. **After the uncoating steps, the viral RNA is released in the cytoplasm. The TRBP-Dicer complex binds to viral and cellular RNAs and cleaves small dsRNAs that inhibit PKR. **B) HIV diverts TRBP from Dicer to avoid the cleavage of its RNA. **The viral RNA released in the cytoplasm binds TRBP, which becomes unavailable for interaction with Dicer. The schematic representation of HIV-1 genomic RNA includes the 5' and 3' TAR RNAs, the RRE RNA, the vsiRNA and other potential stem-loop structures.

2) Does HIV divert TRBP from Dicer to avoid cellular restriction by RNAi? If RNAi is a natural mechanism to restrict HIV replication, HIV must have developed mechanisms to guarantee effective replication. One mechanism is provided by Tat acting as an RNAi suppressor, but it may not be the only pathway. HIV may recruit TRBP and use it for its own benefit to avoid cleavage of its own RNA. TRBP on TAR and RRE RNAs is utilized by the virus to improve its own translation and replication and as a consequence becomes unavailable to bind Dicer and mediate RNAi. In this case, both TRBP and Tat would participate in the inhibition of HIV restriction by RNAi and act in concert to favor viral expression as shown earlier [18,19,23], but by an additional mechanism. Studies on RNAi function and the levels of small RNAs during HIV replication should help to elucidate this hypothesis (Fig. 2B).

Whether HIV co-opts the RNAi pathway for its benefit or whether it diverts TRBP to avoid the cleavage of its RNA remains to be elucidated, but the end result is that the virus proceeds with replication. The final mechanism may come from studies in human cellular models in which the virus replicates poorly. Astrocytes represent such a model, but other models in which either the IFN response or the RNAi mechanism represents major cellular responses, will certainly emerge. TRBP, with its antagonistic properties as an anti-PKR and a pro-Dicer factor will be a key player in the balance between these mechanisms that will lead to viral replication or antiviral immunity.

## Competing interests

The author(s) declare that they have no competing interests.

## Authors' contributions

AG participated to the conception, design and writing of the article. SL participated in the interpretation of data and revision of the manuscript. GC participated in the interpretation of data and drawing of the figures.
